# SARS-CoV-2 anti-spike antibody levels following second dose of ChAdOx1 nCov-19 or BNT162b2 in residents of long-term care facilities in England (VIVALDI)

**DOI:** 10.1093/infdis/jiac146

**Published:** 2022-04-16

**Authors:** Oliver Stirrup, Maria Krutikov, Gokhan Tut, Tom Palmer, David Bone, Rachel Bruton, Chris Fuller, Borscha Azmi, Tara Lancaster, Panagiota Sylla, Nayandeep Kaur, Eliska Spalkova, Christopher Bentley, Umayr Amin, Azar Jadir, Samuel Hulme, Rebecca Giddings, Hadjer Nacer-Laidi, Verity Baynton, Aidan Irwin-Singer, Andrew Hayward, Paul Moss, Andrew Copas, Laura Shallcross

**Affiliations:** 1 UCL Institute of Health Informatics, London, NW1 2DA, United Kingdom; 2 UCL Institute for Global Health, London, WC1N 1EH, United Kingdom; 3 UCL Institute of Epidemiology & Health Care, London, WC1E 7HB, United Kingdom; 4 Institute of Immunology and Immunotherapy, University of Birmingham, Birmingham, B15 2TT, United Kingdom; 5 Health Data Research UK, London, United Kingdom; 6 Department of Health and Social Care, London, SW1H 0EU, United Kingdom

**Keywords:** COVID-19, long-term care facilities, vaccination, antibodies, waning

## Abstract

General population studies have shown strong humoral response following SARS-CoV-2 vaccination with subsequent waning of anti-spike antibody levels. Vaccine-induced immune responses are often attenuated in frail and older populations, but published data are scarce. We measured SARS-CoV-2 anti-spike antibody levels in Long-Term Care Facility residents and staff following second vaccination dose with Oxford-AstraZeneca or Pfizer-BioNTech. Vaccination elicited robust antibody responses in older residents, suggesting comparable levels of vaccine-induced immunity to that in the general population. Antibody levels are higher after Pfizer-BioNTech vaccination but fall more rapidly compared to Oxford-AstraZeneca recipients and are enhanced by prior infection in both groups.

## Introduction

Residents of Long-Term Care Facilities (LTCF) have experienced extremely high rates of SARS-CoV-2 infection and mortality[1]. Since December 2020, LTCF staff and residents in England have been prioritised for vaccination against SARS-CoV-2, with initial roll-out primarily using the mRNA-based BNT162b2 (Pfizer-BioNTech) and adenoviral vector-based ChAdOx1 (Oxford-AstraZeneca) vaccines[2].

Vaccine effectiveness in the general population has been demonstrated for at least six months following second dose administration[3,4]. However, data are limited on the duration and magnitude of protection afforded by vaccination in LTCF residents. Furthermore, LTCF residents are especially vulnerable to severe outcomes following infection due to frailty, high rates of co-morbidity, poorer nutritional status, and age-related dampening of immune responses (immune-senescence) which impact on vaccine-induced immunity[5].

Current SARS-CoV-2 vaccines target the viral spike protein, and anti-spike antibody levels are an important correlate of vaccine efficacy[6]. Early studies are encouraging and suggest robust cellular and humoral responses in the initial months following vaccination amongst LTCF residents, particularly in previously-infected individuals[6,7]. However, studies from the general population have reported waning of antibody titres in the six months following vaccination, particularly in people older than 65 years[8–10]. We investigated quantitative anti-spike antibody titres amongst LTCF staff and residents in England over the first nine months following second vaccination dose.

## Methods

VIVALDI (ISRCTN 14447421) is a prospective cohort study of residents and staff of LTCFs in England[11]. Eligible individuals from participating LTCFs provide written informed consent for study participation and consultees are sought for residents lacking capacity to consent. Participants have undergone up to five rounds of blood sampling at eight-week intervals between 11 June 2020 and 22 October 2021. As part of the national pandemic response, all LTCF staff and residents regularly submit nasopharyngeal swabs for SARS-CoV-2 PCR testing (monthly in residents, weekly in staff) with additional testing during outbreaks[12].

Blood samples undergo SARS-CoV-2 nucleocapsid IgG testing using the Abbott ARCHITECT semi-quantitative immunoassay (Maidenhead, UK). Quantitative antibody titres against SARS-CoV-2 spike and nucleocapsid IgG are measured using the Meso Scale Diagnostics (MSD) V-PLEX COVID-19 Respiratory Panel 2 kit (Rockville, MD, USA). Anti-nucleocapsid antibodies are used to identify immune responses stimulated by prior infection. MSD observations were included from ≥21 days after second vaccine dose administration, corresponding to peak antibody response[4], up until date of third vaccine dose where recorded. Only individuals with data on demographic characteristics and vaccinations were included in this analysis and most could also be linked to full testing history (Appendix S1).

To model post-vaccination MSD assay anti-spike antibody levels, individuals were categorised as either having ‘no evidence of prior infection’ or ‘evidence of prior infection’. The latter group included individuals with at least one record of an active infection defined by PCR or point-of-care lateral flow test (LFT) positivity or hospitalisation with COVID-19 prior to second vaccine dose, and those with presence of anti-nucleocapsid antibodies on either Abbott or MSD assay. To exclude breakthrough infections which may have boosted antibody levels, observations with active infection recorded after second vaccine dose but prior to index date were dropped from analysis, as were observations following post-vaccination anti-nucleocapsid seroconversion.

An index value ≥0.8 defined Abbott anti-nucleocapsid assay positivity[13]. A threshold of 1200 AU/mL was used for MSD anti-nucleocapsid assay, which had a specificity of 96% (48/50) using pre-pandemic blood samples.

VIVALDI has been granted research ethics approval by the South Central-Hampshire B Research Ethics Committee (ref:20/SC/0238).

*Statistical analysis*

Log10-transformed MSD anti-spike levels were modelled using linear mixed effects models. Time was centred at 21 days after second vaccine dose, with random intercept and slope terms for each participant. This approach allows for the analysis of all available data within a single statistical model and can accommodate irregular numbers and timings of measurements for each participant. Intercept terms from the model correspond to estimated peak antibody levels and slope terms correspond to rate of decline over time on the log-scale.

An initial model was fitted with independent effects assumed for vaccine type, sex, staff/resident status and prior SARS-CoV-2 infection, followed by a model with interaction terms between vaccine type and each other variable. A further model was considered with addition of subject-age (centred at 70 years) as a linear predictor of both intercept and slope by vaccine type. Half-life values were calculated based on estimated time to drop in mean log10 antibody level of log10(0.5). Formal sample size calculation was not undertaken.

**<u>Results</u>**

We describe 558 anti-spike antibody (MSD) results from 402 LTCF residents and 759 from 632 staff. 774 people had one observation, 237 had two and 23 had three. Median age was 86 (IQR 78-91) years for residents and 50 (IQR 37-58) years for staff. Samples included in the analysis were collected between 15 March and 22 October 2021. The median time between first and second dose was 74 days (IQR 66-77 days) for residents and 74 days (IQR 63-77 days) for staff (P=0.15 for difference between groups on Mann-Whitney test). Median time from second vaccine dose to blood sample was 136 days (IQR 104-

170, range 21-280). Four observations from four residents and four from three staff were dropped from analysis as they followed detection of an active breakthrough infection. Eight residents and eight staff each had one observation excluded because of indirect evidence of breakthrough infection (i.e., appearance of anti-nucleocapsid antibodies).

The interaction model, allowing different effects by vaccine type, was found to provide better fit to the data than the simpler independent effects model ($P=0.01$, likelihood ratio test (LRT)), and a further improvement was found by adding age as linear predictor of peak antibody levels and slope ($P=0.03$, LRT).

Based on findings from the mixed-effects model, peak antibody titres were greater in Pfizer-B recipients than in Oxford-AZ recipients (×7.9, 95%CI 3.6-17.0; $P<0.01$), although we also observed a steeper annual decline in this group (×0.08 at 12 months *vs* equivalent decline from peak, 0.01-0.72; $P=0.02$) (Table 1, Figure 1). Prior infection with SARS-CoV-2 was associated with higher peak antibody levels and slower decline for both Pfizer-B (peak ×2.8, 1.9-4.1; $P<0.01$) and Oxford-AZ (×4.8, 95%CI 3.2-7.1; $P<0.01$) recipients. Male sex was associated with slightly higher peak in antibody levels for both vaccines (not statistically significant) but steeper decline, particularly for Oxford-AZ recipients. LTCF resident *vs* staff status was not associated with any statistically significant difference in peak antibody level or slope of decline. However, increasing age was associated with lower antibody peak for Oxford-AZ recipients.

'Half-life' estimates of antibody decline were in the range 60-120 days for most subgroups, with values >6 months in female Oxford-AZ recipients with prior infection, but 95% CIs were wide (Table S2).

## Discussion

We present post-vaccination serological data from a large cohort of frail LTCF residents in England, a group in whom published data are scarce. Our findings are broadly consistent with longitudinal studies conducted in the general population and healthcare workers[8,10] which is reassuring given the vulnerability of LTCF residents to SARS-CoV-2 infection.

Consistent with previous studies, we find higher peak antibody titres following vaccination with Pfizer compared to Oxford-AZ[9,10]. Wei et al reported on anti-spike antibody waning in ~100,000 Oxford-AZ and ~55,000 Pfizer-B vaccine recipients, sampled through the Coronavirus Infection Survey (CIS)[10]. For Oxford-AZ they found peak antibody levels were higher in those with prior infection, and slightly lower in males and younger ages. Peak antibody levels were greater in Pfizer-B recipients compared with Oxford-AZ but were lower at older ages and for males[10].

The collection of samples up to 9 months after vaccination allowed us to assess the rate of spike-specific antibody decline from peak value. The mean half-life of antibody decline was reported as 85 days (95%CI 84-86) after Oxford-AZ in the CIS study, and this was increased to 131 days in those with prior infection. They found comparable mean half-life after Pfizer-B of 101 days (100-102) which was extended to 188 days in those with prior infection[10]. Our data also revealed mean half-life in the range 60-120 days but did not uncover significant variation in the rate of antibody decline between LTCF staff and residents. Analysis of >8500 community-dwelling infection-naïve adults also found no difference in rates of waning in donors aged ≥65 years although peak titres declined with age[9].

Our study is consistent in finding higher peak levels and longer half-life associated with prior infection for both vaccine types, and higher peak levels following Pfizer vaccination. However, we find no difference between staff and residents besides a lower peak antibody response in older Oxford-AZ recipients. The level of exposure to infection was much greater in LTCFs than in the community, [1] and those residents who survived infection are likely to have more robust immunological responses to vaccination than their community-dwelling peers who are included in studies of the general population.

Overall, our results are encouraging and add to a body of evidence suggesting strong humoral and cellular responses to vaccination amongst LTCF residents[14].

Our study is limited by a modest sample size, so there is uncertainty regarding the presence and magnitude of observed effects. It is also possible that some individuals labelled as infection-naïve may have waned below the positivity threshold following infection early in the pandemic[15]. To account for this, we used a lower Abbott positivity threshold and included MSD results in defining “prior-exposure”, but we cannot determine the chronology of infection in anti-nucleocapsid antibody positive participants. As the analysis was carried out over a period of relatively low community transmission, it is unlikely that antibody titres have been boosted by undetected breakthrough infections following second dose vaccination.  Finally, we have only described humoral responses to vaccination; analyses in LTCF staff and residents of vaccine-induced cellular immune responses and functional measures of immunity such as neutralization antibody titres are underway by our group and others.

Insights into the magnitude and duration of vaccine-induced immune responses are crucial to inform the timing of booster vaccination, particularly with the emergence of novel variants such as Omicron.  Our findings reveal that current COVID-19 vaccines retain high immunogenicity in the LTCF setting but factors such as peak antibody response and rate of antibody waning, which will be used to guide the need for future vaccinations, are strongly influenced by vaccine regimen and prior infection status. Ongoing assessment of humoral immunity will be important in order to guide introduction of optimal booster regimens that maintain immunity over the longer term.

**Acknowledgements**

We thank the staff and residents in the LTCFs that participated in this study and Mark Marshall at NHS England who pseudonymised the electronic health records. This work is independent research funded by the Department of Health and Social Care (COVID-19 surveillance studies). MK is funded by a

Wellcome Trust Clinical PhD Fellowship (222907/Z/21/Z). LS is funded by a National Institute for Health Research Clinician Scientist Award (CS-2016-007). AH is supported by Health Data Research UK (LOND1), which is funded by the UK Medical Research Council, Engineering and Physical Sciences Research Council, Economic and Social Research Council, Department of Health and Social Care (England), Chief Scientist Office of the Scottish Government Health and Social Care Directorates, Health and Social Care Research and Development Division (Welsh Government), Public Health Agency (Northern Ireland), British Heart Foundation, and Wellcome Trust. The views expressed in this publication are those of the authors and not necessarily those of the NHS, Public Health England, or the Department of Health and Social Care.

LS, TP, AC, and OS report grants from the Department of Health and Social Care during the conduct of the study and LS is a member of the Social Care Working Group, which reports to the Scientific Advisory Group for Emergencies. AIS and VB are employed by the Department of Health and Social Care who funded the study. AH reports funding from the Covid Core Studies Programme is a member of the New and Emerging Respiratory Virus Threats Advisory Group at the Department of Health and Environmental Modelling Group of the Scientific Advisory Group for Emergencies. All other authors declare no competing interests.

## References

1. ECDC. Surveillance data from public online national reports on COVID-19 in long-term care facilities. 2021.

2. GOV.UK. Vaccine update: issue 315, December 2020, COVID-19 special edition - GOV.UK.

3. Andrews N, Tessier E, Stowe J *et al.* Vaccine effectiveness and duration of protection of Comirnaty, Vaxzevria and Spikevax against mild and severe COVID-19 in the UK. *medRxiv* 2021:2021.09.15.21263583.

4. Eyre DW, Lumley SF, Wei J *et al.* Quantitative SARS-CoV-2 anti-spike responses to Pfizer–BioNTech and Oxford–AstraZeneca vaccines by previous infection status. *Clinical Microbiol Infect* 2021;**27**:1516.e7-1516.e14.

5. Castro-Herrera VM, Lown M, Fisk HL *et al.* Relationships Between Age, Frailty, Length of Care Home Residence and Biomarkers of Immunity and Inflammation in Older Care Home Residents in the United Kingdom. *Front Aging* 2021;**0**:2.

6. Feng S, Phillips DJ, White T *et al.* Correlates of protection against symptomatic and asymptomatic SARS-CoV-2 infection. *Nat Med 2021 27:11* 2021;**27**:2032–40.

7. Parry H, Bruton R, Tut G *et al.* Immunogenicity of single vaccination with BNT162b2 or ChAdOx1 nCoV-19 at 5–6 weeks post vaccine in participants aged 80 years or older: an exploratory analysis. *Lancet Healthy Longev* 2021;**2**:e554–60.

8. Levin EG, Lustig Y, Cohen C *et al.* Waning Immune Humoral Response to BNT162b2 Covid-19 Vaccine over 6 Months. *NE JM* 2021;**385**:e84.

9. Aldridge RW, Yavlinsky A, Nguyen V *et al.* Waning of SARS-CoV-2 antibodies targeting the Spike protein in individuals post second dose of ChAdOx1 and BNT162b2 COVID-19 vaccines and risk of breakthrough infections: analysis of the Virus Watch community cohort. *medRxiv* 2021:2021.11.05.21265968.

10. Wei J, Pouwels KB, Stoesser N *et al.* SARS-CoV-2 anti-spike IgG antibody responses after second dose of ChAdOx1 or BNT162b2 and correlates of protection in the UK general population. *medRxiv* 2021:2021.09.13.21263487.

11. Krutikov M, Palmer T, Donaldson A *et al.* Study Protocol: Understanding SARS-Cov-2 infection, immunity and its duration in care home residents and staff in England (VIVALDI). *Wellcome Open Res* 2020;**5**:232.

12. GOV.UK. Coronavirus (COVID-19) testing in adult care homes. 2021.

13. Ainsworth M, Andersson M, Auckland K *et al.* Performance characteristics of five immunoassays for SARS-CoV-2: a head-to-head benchmark comparison. *The Lancet Infect Dis* 2020;**20**:1390–400.

14. Tut G, Lancaster T, Krutikov M *et al.* Profile of humoral and cellular immune responses to single doses of BNT162b2 or ChAdOx1 nCoV-19 vaccines in residents and staff within residential care homes (VIVALDI): an observational study. *Lancet Healthy Longev* 2021;**2**:e544–53.

15. Krutikov M, Palmer T, Tut G *et al.* Prevalence and duration of detectable SARS-CoV-2 nucleocapsid antibodies in staff and residents of long-term care facilities over the first year of the pandemic (VIVALDI study): prospective cohort study in England. *Lancet Healthy Longev* 2021;**0**:2021.

**Table 1** Regression coefficients from final statistical mixed-effects model for anti-spike antibody levels from 21 days following second vaccine dose, fitted to log10-transformed data

| | *n, n* (%*) or median (IQR) | ***Intercept† (95%CI); P*** | ***Slope (95% CI); P [annual change]*** |
|---|---|---|---|
| Reference coefficients‡ | | 4.12 (3.86 to 4.38) | -0.67 (-1.48 to 0.14) |
| *Oxford-AZ recipients* | 493 | ***Difference in intercept (95%CI)#; P*** | ***Difference in slope (95%CI)**; P*** |
| Prior infection (yes *vs* no) | 246 (49.9) | 0.68 (0.5 to 0.85); <0.01 | 0.50 (-0.01 to 1.01); 0.06 |
| LTCF resident (*vs* staff) | 251 (50.9) | 0.22 (-0.14 to 0.59); 0.23 | -0.45 (-1.58 to 0.67); 0.43 |
| Male (*vs* female) | 105 (21.3) | 0.17 (-0.05 to 0.39); 0.13 | -0.69 (-1.32 to -0.05); 0.03 |
| Age (per 10y greater than 70) | 67 (48–87) | -0.10 (-0.18 to -0.02); 0.01 | 0.16 (-0.09 to 0.42); 0.20 |
| *Pfizer-B. recipients* | 534 | ***Difference in intercept (95%CI)#; P*** | ***Difference in slope (95%CI)**; P*** |

| | | | |
|---|---|---|---|
| Difference *vs* Oxford-AZ¶ | | 0.90 (0.56 to 1.23); <0.01 | -1.09 (-2.04 to -0.14); 0.02 |
| Prior infection (yes *vs* no) | 306 (57.3) | 0.44 (0.27 to 0.61); <0.01 | 0.43 (0.01 to 0.85); 0.04 |
| LTCF resident (*vs* staff) | 147 (27.5) | -0.05 (-0.36 to 0.26); 0.74 | 0.06 (-0.7 to 0.82); 0.87 |
| Male (*vs* female) | 94 (17.6) | 0.11 (-0.1 to 0.31); 0.31 | -0.23 (-0.72 to 0.26); 0.36 |
| Age (per 10y greater than 70) | 56 (44–71) | -0.01 (-0.08 to 0.06); 0.76 | -0.06 (-0.23 to 0.11); 0.49 |

*LTCF, long-term care facility.*

**% calculated using number with same vaccine type as denominator.*

*†Representing average peak value at 21 days after second vaccine dose.*

*‡Values for Oxford-AZ recipient female staff member at 70 years of age without prior infection. ¶Taken alone, represents the difference for female staff member at 70 years of age without prior infection.*

*#10^x gives multiplicative difference in intercept associated with each factor.*

***10^x gives multiplicative difference in value at 12 months from peak level.*

**Figure 1:** Log-transformed MSD values for anti-spike antibody levels in relation to the time from second vaccine dose, divided by vaccine type and staff/resident status, and colour-coded by prior infection category (red: evidence of prior infection; green: no evidence of prior infection). Individual observations are shown as dots, with those from the same person linked by lines. The bold straight lines show regression fits from a statistical model (omitting age and sex) to estimate trends in each group.

## Data sharing

De-identified test results and limited metadata will be made available for use by researchers in future studies, subject to appropriate research ethical approvals once the VIVALDI study cohort has been finalised. These datasets will be accessible via the Health Data Research UK Gateway.

**Figure 1**
**165x128 mm (9.9 x  DPI)**

## Supplementary Material

jiac146_Supplementary_Data

