# Severe Acute Respiratory Syndrome Coronavirus 2 Anti-Spike Antibody Levels Following Second Dose of ChAdOx1 nCov-19 or BNT162b2 Vaccine in Residents of Long-term Care Facilities in England (VIVALDI)

**DOI:** 10.1093/infdis/jiac146

**Published:** 2022-04-16

**Authors:** Oliver Stirrup, Maria Krutikov, Gokhan Tut, Tom Palmer, David Bone, Rachel Bruton, Chris Fuller, Borscha Azmi, Tara Lancaster, Panagiota Sylla, Nayandeep Kaur, Eliska Spalkova, Christopher Bentley, Umayr Amin, Azar Jadir, Samuel Hulme, Rebecca Giddings, Hadjer Nacer-Laidi, Verity Baynton, Aidan Irwin-Singer, Andrew Hayward, Paul Moss, Andrew Copas, Laura Shallcross

**Affiliations:** Institute for Global Health, University College London, United Kingdom; Institute of Health Informatics, University College London, United Kingdom; Institute of Immunology and Immunotherapy, University of Birmingham, United Kingdom; Institute for Global Health, University College London, United Kingdom; Institute of Immunology and Immunotherapy, University of Birmingham, United Kingdom; Institute of Immunology and Immunotherapy, University of Birmingham, United Kingdom; Institute of Health Informatics, University College London, United Kingdom; Institute of Health Informatics, University College London, United Kingdom; Institute of Immunology and Immunotherapy, University of Birmingham, United Kingdom; Institute of Immunology and Immunotherapy, University of Birmingham, United Kingdom; Institute of Immunology and Immunotherapy, University of Birmingham, United Kingdom; Institute of Immunology and Immunotherapy, University of Birmingham, United Kingdom; Institute of Immunology and Immunotherapy, University of Birmingham, United Kingdom; Institute of Immunology and Immunotherapy, University of Birmingham, United Kingdom; Institute of Immunology and Immunotherapy, University of Birmingham, United Kingdom; Institute of Immunology and Immunotherapy, University of Birmingham, United Kingdom; Institute of Health Informatics, University College London, United Kingdom; Institute of Health Informatics, University College London, United Kingdom; Department of Health and Social Care, London, United Kingdom; Department of Health and Social Care, London, United Kingdom; Institute of Epidemiology and Health Care, University College London, United Kingdom; Health Data Research UK, London, United Kingdom; Institute of Immunology and Immunotherapy, University of Birmingham, United Kingdom; Institute for Global Health, University College London, United Kingdom; Institute of Health Informatics, University College London, United Kingdom

**Keywords:** COVID-19, long-term care facilities, vaccination, antibodies, waning

## Abstract

General population studies have shown strong humoral response following severe acute respiratory syndrome coronavirus 2 (SARS-CoV-2) vaccination with subsequent waning of anti-spike antibody levels. Vaccine-induced immune responses are often attenuated in frail and older populations, but published data are scarce. We measured SARS-CoV-2 anti-spike antibody levels in long-term care facility residents and staff following a second vaccination dose with Oxford-AstraZeneca or Pfizer-BioNTech. Vaccination elicited robust antibody responses in older residents, suggesting comparable levels of vaccine-induced immunity to that in the general population. Antibody levels are higher after Pfizer-BioNTech vaccination but fall more rapidly compared to Oxford-AstraZeneca recipients and are enhanced by prior infection in both groups.

## Teaser

Antibody responses to vaccination in older residents of long-term care facilities are comparable to those in the general population. Although antibody levels are initially greater, they fall more rapidly following Pfizer-BioNTech vaccination than Oxford-AstraZeneca. Prior infection enhances responses in both groups.

Residents of long-term care facilities (LTCF) have experienced extremely high rates of severe acute respiratory syndrome coronavirus 2 (SARS-CoV-2) infection and mortality [1]. Since December 2020, LTCF staff and residents in England have been prioritized for vaccination against SARS-CoV-2, with initial rollout primarily using the messenger RNA–based BNT162b2 (Pfizer-BioNTech) and adenoviral vector–based ChAdOx1 (Oxford-AstraZeneca [Oxford-AZ]) vaccines [2].

Vaccine effectiveness in the general population has been demonstrated for at least 6 months following second dose administration [3, 4]. However, data are limited on the duration and magnitude of protection afforded by vaccination in LTCF residents. Furthermore, LTCF residents are especially vulnerable to severe outcomes following infection due to frailty, high rates of comorbidity, poorer nutritional status, and age-related dampening of immune responses (immunosenescence), which impact vaccine-induced immunity [5].

Current SARS-CoV-2 vaccines target the viral spike protein, and anti-spike antibody levels are an important correlate of vaccine efficacy [6]. Early studies are encouraging and suggest robust cellular and humoral responses in the initial months following vaccination among LTCF residents, particularly in previously infected individuals [6, 7]. However, studies from the general population have reported waning of antibody titers in the 6 months following vaccination, particularly in people older than 65 years [8–10]. We investigated quantitative anti-spike antibody titers among LTCF staff and residents in England over the first 9 months following second vaccination dose.

## METHODS

XXX (VIVALDI; ISRCTN 14447421) is a prospective cohort study of residents and staff of LTCFs in England [11]. Eligible individuals from participating LTCFs provide written informed consent for study participation and consultees are sought for residents lacking capacity to consent. Participants have undergone up to 5 rounds of blood sampling at 8-week intervals between 11 June 2020 and 22 October 2021. As part of the national pandemic response, all LTCF staff and residents regularly submit nasopharyngeal swabs for SARS-CoV-2 polymerase chain reaction (PCR) testing (monthly in residents, weekly in staff) with additional testing during outbreaks [12].

Blood samples undergo SARS-CoV-2 nucleocapsid immunoglobulin G (IgG) testing using the Abbott ARCHITECT semi-quantitative immunoassay (Maidenhead, United Kingdom). Quantitative antibody titers against SARS-CoV-2 spike and nucleocapsid IgG are measured using the Meso Scale Diagnostics (MSD) V-PLEX COVID-19 (coronavirus disease 2019) Respiratory Panel 2 kit (Rockville, Maryland). Anti-nucleocapsid antibodies are used to identify immune responses stimulated by prior infection. MSD observations were included from ≥21 days after second vaccine dose administration, corresponding to peak antibody response [4], up until date of third vaccine dose where recorded. Only individuals with data on demographic characteristics and vaccinations were included in this analysis and most could also be linked to full testing history (Supplementary Appendix 1).

To model postvaccination MSD assay anti-spike antibody levels, individuals were categorized as either having no evidence of prior infection or evidence of prior infection. The latter group included individuals with at least 1 record of an active infection defined by PCR or point-of-care lateral flow test positivity or hospitalization with COVID-19 prior to second vaccine dose, and those with presence of anti-nucleocapsid antibodies on either Abbott or MSD assay. To exclude breakthrough infections, which may have boosted antibody levels, observations with active infection recorded after second vaccine dose but prior to index date were dropped from analysis, as were observations following postvaccination anti-nucleocapsid seroconversion.

An index value ≥0.8 defined the Abbott anti-nucleocapsid assay positivity [13]. A threshold of 1200 AU/mL was used for the MSD anti-nucleocapsid assay, which had a specificity of 96% (48/50) using prepandemic blood samples.

VIVALDI has been granted research ethics approval by the South Central-Hampshire B Research Ethics Committee (reference number 20/SC/0238).

### Statistical Analysis

Log_10_-transformed MSD anti-spike levels were modeled using linear mixed-effects models. Time was centered at 21 days after second vaccine dose, with random intercept and slope terms for each participant. This approach allows for the analysis of all available data within a single statistical model and can accommodate irregular numbers and timings of measurements for each participant. Intercept terms from the model correspond to estimated peak antibody levels, and slope terms correspond to rate of decline over time on the log scale.

An initial model was fitted with independent effects assumed for vaccine type, sex, staff/resident status, and prior SARS-CoV-2 infection, followed by a model with interaction terms between vaccine type and each other variable. A further model was considered with addition of subject age (centered at 70 years) as a linear predictor of both intercept and slope by vaccine type. Half-life values were calculated based on estimated time to drop in mean log_10_ antibody level of log_10_(0.5). Formal sample size calculation was not undertaken.

## RESULTS

We describe 558 anti-spike antibody (MSD) results from 402 LTCF residents and 759 from 632 staff. A total of 774 people had 1 observation, 237 had 2 observations, and 23 had 3 observations. Median age was 86 years (interquartile range [IQR], 78–91 years) for residents and 50 years (IQR, 37–58 years) for staff. Samples included in the analysis were collected between 15 March and 22 October 2021. The median time between first and second dose was 74 days (IQR, 66–77 days) for residents and 74 days (IQR, 63–77 days) for staff (*P* = .15 for difference between groups on Mann–Whitney test). Median time from second vaccine dose to blood sample was 136 days (IQR, 104–170 days; range, 21–280 days). Four observations from 4 residents and 4 from 3 staff were dropped from analysis as they followed detection of an active breakthrough infection. Eight residents and 8 staff each had 1 observation excluded because of indirect evidence of breakthrough infection (ie, appearance of anti-nucleocapsid antibodies).

The interaction model, allowing different effects by vaccine type, was found to provide better fit to the data than the simpler independent effects model (*P* = .01, likelihood ratio test [LRT]), and a further improvement was found by adding age as linear predictor of peak antibody levels and slope (*P* = .03, LRT).

**Figure 1.**
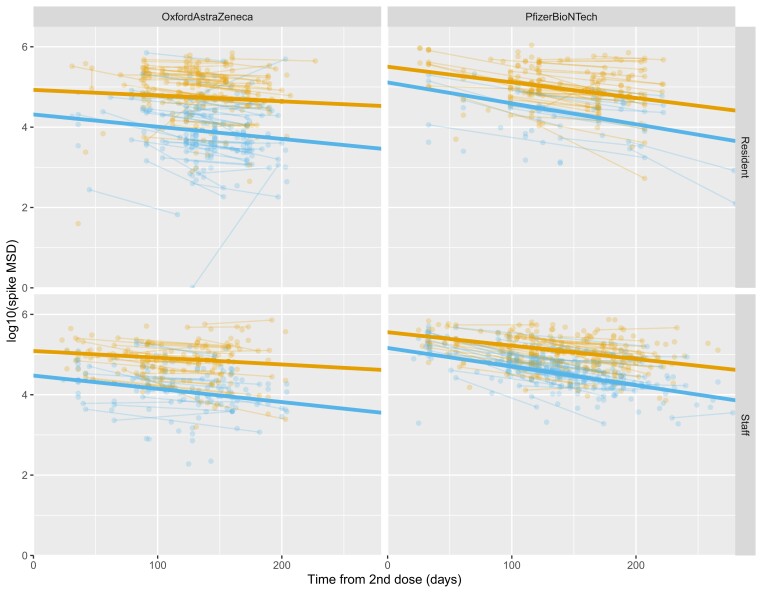
Log-transformed Meso Scale Diagnostics (MSD) values for anti-spike antibody levels in relation to the time from second vaccine dose, divided by vaccine type and staff/resident status, and color-coded by prior infection category (orange: evidence of prior infection; blue: no evidence of prior infection). Individual observations are shown as dots, with those from the same person linked by lines. The bold straight lines show regression fits from a statistical model (omitting age and sex) to estimate trends in each group.

Based on findings from the mixed-effects model, peak antibody titers were greater in Pfizer-BioNTech recipients than in Oxford-AZ recipients (7.9 times [95% confidence interval {CI}, 3.6–17.0]; *P* < .01), although we also observed a steeper annual decline in this group (0.08 times at 12 months vs equivalent decline from peak [95% CI, .01–.72]; *P* = .02) (Table 1, Figure 1). Prior infection with SARS-CoV-2 was associated with higher peak antibody levels and slower decline for both Pfizer-BioNTech (peak, 2.8 times [95% CI, 1.9–4.1]; *P* < .01) and Oxford-AZ (4.8 times [95% CI, 3.2–7.1]; *P* < .01) recipients. Male sex was associated with slightly higher peak in antibody levels for both vaccines (not statistically significant) but steeper decline, particularly for Oxford-AZ recipients. LTCF resident *vs* staff status was not associated with any statistically significant difference in peak antibody level or slope of decline. However, increasing age was associated with lower antibody peak for Oxford-AZ recipients.

**Table 1.** Regression Coefficients From Final Statistical Mixed-Effects Model for Anti-Spike Antibody Levels From 21 Days Following Second Vaccine Dose, Fitted to Log_10_-Transformed Data

Characteristic	No., No. (%^a^), or Median (IQR)	Intercept^b^ (95% CI)	*P* Value	Slope (95% CI)	*P* Value (Annual Change)
Reference coefficients^c^		4.12 (3.86 to 4.38)		−0.67 (−1.48 to .14)	
Oxford-AZ recipients	493	Difference in Intercept (95% CI)^d^		Difference in Slope (95% CI)^e^	
Prior infection (yes vs no)	246 (49.9)	0.68 (.5 to .85)	<.01	0.50 (−.01 to 1.01)	.06
LTCF resident (vs staff)	251 (50.9)	0.22 (−.14 to .59)	.23	−0.45 (−1.58 to .67)	.43
Male (vs female)	105 (21.3)	0.17 (−.05 to .39)	.13	−0.69 (−1.32 to −.05)	.03
Age (per 10 y greater than 70)	67 (48 to 87)	−0.10 (−.18 to −.02)	.01	0.16 (−.09 to .42)	.20
Pfizer-BioNTech recipients	534				
Difference vs Oxford-AZ^f^		0.90 (.56 to 1.23)	<.01	−1.09 (−2.04 to −.14)	.02
Prior infection (yes vs no)	306 (57.3)	0.44 (.27 to .61)	<.01	0.43 (.01 to .85)	.04
LTCF resident (vs staff)	147 (27.5)	−0.05 (−.36 to .26)	.74	0.06 (−.7 to .82)	.87
Male (vs female)	94 (17.6)	0.11 (−.1 to .31)	.31	−0.23 (−.72 to .26)	.36
Age (per 10 y greater than 70)	56 (44 to 71)	−0.01 (−.08 to .06)	.76	−0.06 (−.23 to .11)	.49

Abbreviations: AZ, AstraZeneca; CI, confidence interval; IQR, interquartile range; LTCF, long-term care facility.

a Percentage calculated using number with same vaccine type as denominator.

b Representing average peak value at 21 days after second vaccine dose.

c Values for Oxford-AZ recipient female staff member at 70 years of age without prior infection.

d 10^x^ gives multiplicative difference in intercept associated with each factor.

e 10^x^ gives multiplicative difference in value at 12 months from peak level.

f Taken alone, represents the difference for female staff member at 70 years of age without prior infection.

“Half-life” estimates of antibody decline were in the range of 60–120 days for most subgroups, with values >6 months in female Oxford-AZ recipients with prior infection, but 95% CIs were wide (Supplementary Table 2).

## DISCUSSION

We present postvaccination serological data from a large cohort of frail LTCF residents in England, a group in whom published data are scarce. Our findings are broadly consistent with longitudinal studies conducted in the general population and healthcare workers [8, 10] which is reassuring given the vulnerability of LTCF residents to SARS-CoV-2 infection.

Consistent with previous studies, we find higher peak antibody titers following vaccination with Pfizer-BioNTech compared to Oxford-AZ [9, 10]. Wei et al reported on anti-spike antibody waning in approximately 100 000 Oxford-AZ and approximately 55 000 Pfizer-BioNTech vaccine recipients, sampled through the Coronavirus Infection Survey (CIS) [10]. For Oxford-AZ, they found that peak antibody levels were higher in those with prior infection, and slightly lower in males and younger ages. Peak antibody levels were greater in Pfizer-BioNTech recipients compared with Oxford-AZ but were lower at older ages and for males [10].

The collection of samples up to 9 months after vaccination allowed us to assess the rate of spike-specific antibody decline from peak value. The mean half-life of antibody decline was reported as 85 days (95% CI, 84–86 days) after Oxford-AZ in the CIS study, and this was increased to 131 days in those with prior infection. They found a comparable mean half-life after Pfizer-BioNTech of 101 days (95% CI, 100–102), which was extended to 188 days in those with prior infection [10]. Our data also revealed a mean half-life in the range of 60–120 days but did not uncover significant variation in the rate of antibody decline between LTCF staff and residents. Analysis of >8500 community-dwelling infection-naive adults also found no difference in rates of waning in donors aged ≥65 years, although peak titers declined with age [9].

Our study is consistent in finding higher peak levels and longer half-life associated with prior infection for both vaccine types, and higher peak levels following Pfizer-BioNTech vaccination. However, we find no difference between staff and residents besides a lower peak antibody response in older Oxford-AZ recipients. The level of exposure to infection was much greater in LTCFs than in the community [1], and those residents who survived infection are likely to have more robust immunological responses to vaccination than their community-dwelling peers who are included in studies of the general population. Overall, our results are encouraging and add to a body of evidence suggesting strong humoral and cellular responses to vaccination among LTCF residents [14].

Our study is limited by a modest sample size, so there is uncertainty regarding the presence and magnitude of observed effects. It is also possible that some individuals labeled as infection-naive may have waned below the positivity threshold following infection early in the pandemic [15]. To account for this, we used a lower Abbott positivity threshold and included MSD results in defining prior exposure, but we cannot determine the chronology of infection in anti-nucleocapsid antibody–positive participants. As the analysis was carried out over a period of relatively low community transmission, it is unlikely that antibody titers had been boosted by undetected breakthrough infections following second-dose vaccination. Finally, we have only described humoral responses to vaccination; analyses in LTCF staff and residents of vaccine-induced cellular immune responses and functional measures of immunity such as neutralization antibody titers are underway by our group and others.

Insights into the magnitude and duration of vaccine-induced immune responses are crucial to inform the timing of booster vaccination, particularly with the emergence of novel variants such as Omicron. Our findings reveal that current COVID-19 vaccines retain high immunogenicity in the LTCF setting but factors such as peak antibody response and rate of antibody waning, which will be used to guide the need for future vaccinations, are strongly influenced by vaccine regimen and prior infection status. Ongoing assessment of humoral immunity will be important to guide introduction of optimal booster regimens that maintain immunity over the longer term.

## Supplementary Data

Supplementary materials are available at *The Journal of Infectious Diseases* online (http://jid.oxfordjournals.org/). Supplementary materials consist of data provided by the author that are published to benefit the reader. The posted materials are not copyedited. The contents of all supplementary data are the sole responsibility of the authors. Questions or messages regarding errors should be addressed to the author.

## Notes

***Acknowledgments.*** We thank the staff and residents in the long-term care facilities (LTCFs) that participated in this study and Mark Marshall at the National Health Service (NHS) England who pseudonymized the electronic health records.

***Data sharing.*** De-identified test results and limited metadata will be made available for use by researchers in future studies, subject to appropriate research ethical approvals once the VIVALDI study cohort has been finalized. These datasets will be accessible via the Health Data Research UK Gateway.

***Disclaimer.*** The views expressed in this publication are those of the authors and not necessarily those of the NHS, Public Health England, or the Department of Health and Social Care. Funding to pay the Open Access publication charges for this article was provided by the Wellcome Trust as part of M.K.'s fellowship.

***Financial support.*** This work is independent research funded by the Department of Health and Social Care (COVID-19 surveillance studies). M. K. is funded by a Wellcome Trust Clinical PhD Fellowship (222907/Z/21/Z). L. S. is funded by a National Institute for Health Research Clinician Scientist Award (CS-2016-007). A. H. is supported by Health Data Research UK (LOND1), which is funded by the UK Medical Research Council, Engineering and Physical Sciences Research Council, Economic and Social Research Council, Department of Health and Social Care (England), Chief Scientist Office of the Scottish Government Health and Social Care Directorates, Health and Social Care Research and Development Division (Welsh Government), Public Health Agency (Northern Ireland), British Heart Foundation, and Wellcome Trust.

## Supplementary Material

jiac146_Supplementary_Data

## References

[1] European Centre for Disease Prevention and Control . Surveillance data from public online national reports on COVID-19 in long-term care facilities. 2021. https://www.ecdc.europa.eu/en/all-topics-z/coronavirus/threats-and-outbreaks/covid-19/prevention-and-control/LTCF-data2021. Accessed 21 December 2021.

[2] Public Health England . Vaccine update: issue 315, December 2020, COVID-19 special edition. 2020. https://www.gov.uk/government/publications/vaccine-update-issue-315-december-2020-covid-19-special-edition/vaccine-update-issue-315-december-2020-covid-19-special-edition. Accessed 21 December 2021.

[3] Andrews N , Tessier E, Stowe J, et al Vaccine effectiveness and duration of protection of Comirnaty, Vaxzevria and Spikevax against mild and severe COVID-19 in the UK. medRxiv [Preprint]. Posted online 6 October 2021. doi:10.1101/2021.09.15.21263583.

[4] Eyre DW , Lumley SF, Wei J, et al Quantitative SARS-CoV-2 anti-spike responses to Pfizer–BioNTech and Oxford–AstraZeneca vaccines by previous infection status. Clin Microbiol Infect 2021; 27:1516.e7–14. 10.1016/j.cmi.2021.05.041 PMC8180449 34111577

[5] Castro-Herrera VM , Lown M, Fisk HL, et al Relationships between age, frailty, length of care home residence and biomarkers of immunity and inflammation in older care home residents in the United Kingdom. Front Aging 2021; 2:599084. 35821989 10.3389/fragi.2021.599084 PMC9261419

[6] Feng S , Phillips DJ, White T, et al Correlates of protection against symptomatic and asymptomatic SARS-CoV-2 infection. Nat Med 2021; 27:2032–40. 34588689 10.1038/s41591-021-01540-1 PMC8604724

[7] Parry H , Bruton R, Tut G, et al Immunogenicity of single vaccination with BNT162b2 or ChAdOx1 nCoV-19 at 5–6 weeks post vaccine in participants aged 80 years or older: an exploratory analysis. Lancet Healthy Longev 2021; 2:e554–60. 34401865 10.1016/S2666-7568(21)00169-0 PMC8357462

[8] Levin EG , Lustig Y, Cohen C, et al Waning immune humoral response to BNT162b2 covid-19 vaccine over 6 months. N Engl J Med 2021; 385:e84. 34614326 10.1056/NEJMoa2114583 PMC8522797

[9] Aldridge RW , Yavlinsky A, Nguyen V, et al Waning of SARS-CoV-2 antibodies targeting the Spike protein in individuals post second dose of ChAdOx1 and BNT162b2 COVID-19 vaccines and risk of breakthrough infections: analysis of the Virus Watch community cohort. medRxiv [Preprint]. Posted online 9 November 2021. doi:10.1101/2021.11.05.21265968.

[10] Wei J , Pouwels KB, Stoesser N, et al SARS-CoV-2 anti-spike IgG antibody responses after second dose of ChAdOx1 or BNT162b2 and correlates of protection in the UK general population. medRxiv [Preprint]. Posted online 14 January 2022.

[11] Krutikov M , Palmer T, Donaldson A, et al Study protocol: understanding SARS-Cov-2 infection, immunity and its duration in care home residents and staff in England (VIVALDI). Wellcome Open Res 2020; 5:232. 33564722 10.12688/wellcomeopenres.16193.1 PMC7851710

[12] UK Health Security Agency . Coronavirus (COVID-19) testing in adult care homes. 2021. https://www.gov.uk/government/publications/coronavirus-covid-19-testing-in-adult-care-homes. Accessed 21 December 2021.

[13] Ainsworth M , Andersson M, Auckland K, et al Performance characteristics of five immunoassays for SARS-CoV-2: a head-to-head benchmark comparison. Lancet Infect Dis 2020; 20:1390–400. 32979318 10.1016/S1473-3099(20)30634-4 PMC7511171

[14] Tut G , Lancaster T, Krutikov M, et al Profile of humoral and cellular immune responses to single doses of BNT162b2 or ChAdOx1 nCoV-19 vaccines in residents and staff within residential care homes (VIVALDI): an observational study. Lancet Healthy Longev 2021; 2:e544–53. 34430954 10.1016/S2666-7568(21)00168-9 PMC8376213

[15] Krutikov M , Palmer T, Tut G, et al Prevalence and duration of detectable SARS-CoV-2 nucleocapsid antibodies in staff and residents of long-term care facilities over the first year of the pandemic (VIVALDI study): prospective cohort study in England. Lancet Healthy Longev 2022; 3:e13–21. 34935001 10.1016/S2666-7568(21)00282-8 PMC8676418

